# A Pfs48/45-based vaccine to block *Plasmodium falciparum* transmission: phase 1, open-label, clinical trial

**DOI:** 10.1186/s12916-024-03379-y

**Published:** 2024-04-23

**Authors:** M. Alkema, M. J. Smit, C. Marin-Mogollon, K. Totté, K. Teelen, G. J. van Gemert, M. van de Vegte-Bolmer, B. G. Mordmüller, J. M. Reimer, K. L. Lövgren-Bengtsson, R. W. Sauerwein, T. Bousema, J. Plieskatt, M. Theisen, M. M. Jore, M. B. B. McCall

**Affiliations:** 1 Department of Medical Microbiology, Radboud University Medical Center, Nijmegen, the Netherlands; 2 Novavax AB, Uppsala, Sweden; 3 Present Address: TropIQ Health Sciences, Nijmegen, the Netherlands; 4 Department for Congenital Disorders, Statens Serum Institut, Copenhagen, Denmark; 5 Centre for Medical Parasitology at Department of Immunology and Microbiology, University of Copenhagen, Copenhagen, Denmark

**Keywords:** *Plasmodium falciparum*, Vaccine, Transmission-blocking, Pfs48/45

## Abstract

**Background:**

The stalling global progress in malaria control highlights the need for novel tools for malaria elimination, including transmission-blocking vaccines. Transmission-blocking vaccines aim to induce human antibodies that block parasite development in the mosquito and mosquitoes becoming infectious. The Pfs48/45 protein is a leading *Plasmodium falciparum* transmission-blocking vaccine candidate. The R0.6C fusion protein, consisting of Pfs48/45 domain 3 (6C) and the N-terminal region of *P. falciparum* glutamate-rich protein (R0), has previously been produced in *Lactococcus lactis* and elicited functional antibodies in rodents. Here, we assess the safety and transmission-reducing efficacy of R0.6C adsorbed to aluminium hydroxide with and without Matrix-M™ adjuvant in humans.

**Methods:**

In this first-in-human, open-label clinical trial, malaria-naïve adults, aged 18–55 years, were recruited at the Radboudumc in Nijmegen, the Netherlands. Participants received four intramuscular vaccinations on days 0, 28, 56 and 168 with either 30 µg or 100 µg of R0.6C and were randomised for the allocation of one of the two different adjuvant combinations: aluminium hydroxide alone, or aluminium hydroxide combined with Matrix-M1™ adjuvant. Adverse events were recorded from inclusion until 84 days after the fourth vaccination. Anti-R0.6C and anti-6C IgG titres were measured by enzyme-linked immunosorbent assay. Transmission-reducing activity of participants’ serum and purified vaccine-specific immunoglobulin G was assessed by standard membrane feeding assays using laboratory-reared *Anopheles stephensi* mosquitoes and cultured *P. falciparum* gametocytes.

**Results:**

Thirty-one participants completed four vaccinations and were included in the analysis. Administration of all doses was safe and well-tolerated, with one related grade 3 adverse event (transient fever) and no serious adverse events occurring. Anti-R0.6C and anti-6C IgG titres were similar between the 30 and 100 µg R0.6C arms, but higher in Matrix-M1™ arms. Neat participant sera did not induce significant transmission-reducing activity in mosquito feeding experiments, but concentrated vaccine-specific IgGs purified from sera collected two weeks after the fourth vaccination achieved up to 99% transmission-reducing activity.

**Conclusions:**

R0.6C/aluminium hydroxide with or without Matrix-M1™ is safe, immunogenic and induces functional Pfs48/45-specific transmission-blocking antibodies, albeit at insufficient serum concentrations to result in transmission reduction by neat serum. Future work should focus on identifying alternative vaccine formulations or regimens that enhance functional antibody responses.

**Trial registration:**

The trial is registered with ClinicalTrials.gov under identifier NCT04862416.

**Supplementary Information:**

The online version contains supplementary material available at 10.1186/s12916-024-03379-y.

## Background

With almost 250 million infections and approximately 600 thousand deaths per year, malaria remains a global health priority. The renewed focus on malaria elimination has increased the priority of research into interventions to block malaria transmission [1, 2]. By interrupting the highly efficient transmission of malaria parasites by mosquito vectors from infected to susceptible individuals, a significant reduction in the number of secondary infections can be achieved, resulting in an overall reduction in disease and mortality [3]. Malaria transmission-blocking vaccines (TBVs) aim to interrupt transmission to, or the development of parasites in, the mosquito vector by vaccination of the human host [4]. Deployment of TBVs is considered to be an efficient element in an integrated program of anti-malarial interventions, aiming to reduce the overall malaria burden, contain drug resistance, and move towards malaria elimination [2, 5].

Transmission of malaria is dependent on the uptake of male and female gametocytes, the sexual reproductive forms of the *Plasmodium* parasite, in the mosquito blood meal and their subsequent fertilisation in the mosquito midgut. Transmission-blocking vaccine candidate antigens are expressed during gamete stages (Pfs48/45 and Pfs230), zygote and ookinete stages (Pfs25 and Pfs28), or alternatively by the mosquito midgut (AnAPN1) (reviewed in [6]). Antibodies against these antigens can interfere with parasite development in the mosquito when taken up in a bloodmeal, preventing onward transmission. Until now, only Pfs25 and Pfs230 have reached clinical evaluation of which the latter has recently shown promising, durable functional activity in Malian adults [7, 8]. The sexual stage Pfs48/45 antigen has a critical role in parasite fertilisation and is a lead candidate for a *P. falciparum* TBV as naturally-acquired human antibodies that target this protein can exert potent transmission-reducing activity (TRA) [9–11]. Pfs48/45 is expressed by gametocytes, but while these still reside within the human host, this protein remains hidden from the immune system inside the host red blood cell, and therefore cannot be targeted by antibodies. When a mosquito takes a gametocyte-containing bloodmeal, however, the parasite emerges from the red blood cell and Pfs48/45 becomes accessible to antibodies present in the blood meal. Antibodies targeting the C-terminal domain of P48/45 (D3 or 6C) can prevent oocyst and ultimately sporozoite development [10, 12, 13]. Recent findings show that administration of TB31F, a monoclonal antibody targeting the 6C region of Pfs48/45, to malaria naïve trial participants resulted in high-level TRA of their sera in standard membrane feeding assays (SMFA) [14]. In such assays, cultured *P. falciparum* gametocytes are fed to laboratory-reared *Anopheles* mosquitoes in the presence or absence of test sera or antibodies. TRA is expressed as the reduction of oocyst count in mosquitoes fed on gametocytes in the presence of the test serum compared to a non-serum control.

The R0.6C fusion protein consists of the C-terminal 6-cysteine domain of Pfs48/45 (6C or D3) and the N-terminal region of asexual stage *P. falciparum* glutamate-rich protein GLURP (R0) produced in *Lactococcus lactis* [15]. Preclinical immunisation studies with adjuvants approved for use in humans revealed that R0.6C, when formulated with either aluminium hydroxide or Matrix-M1™ alone, induced modest TRA in SMFA. The addition of Matrix-M1™ to R0.6C adsorbed on aluminium hydroxide substantially increased immunogenicity relative to R0.6C administration with either adjuvant alone, resulting in strong TRA [16]. Here, we report the safety, tolerability, immunogenicity and TRA of R0.6C/aluminium hydroxide without or with Matrix-M1™, the first Pfs48/45-based *Plasmodium falciparum* TBV to be assessed in humans.

## Methods

### Study setting and population

This first-in-human, open-label, randomised trial was conducted at the Radboud University Medical Center (Nijmegen, the Netherlands). The study population comprised healthy, male and female malaria-naïve adults aged 18–55 years. All participants provided written informed consent prior to screening. Screening procedures included medical history, physical examination, urine toxicology screening, a pregnancy test for participants of childbearing potential and blood collection for routine clinical laboratory testing of biochemical and haematological parameters, as well as HIV, hepatitis B, and hepatitis C serological screening. The trial protocol (research file number NL7666.000.21) received ethics and regulatory approval by the Netherlands’ Central Committee on Research Involving Human Subjects (CCMO) and a positive marginal review for research with a medicinal product by the national competent authority (Ministry of Health, Welfare and Sport). The trial was registered at ClinicalTrials.gov, identifier NCT04862416 and EudraCT, identifier 2021–000017-17.

### Study product

The R0.6C fusion protein is a chimaera consisting of the 6-cysteine C-terminal fragment of Pfs48/45 (D3, 6C) and the N-terminal region of asexual stage glutamate-rich protein GLURP (R0) produced in *Lactococcus lactis* [15, 17, 18]. The recombinant R0.6C protein is formulated in 10 mM HEPES, 2.5% glucose, 0.5 mM EDTA, 155 mM NaCl and absorbed to 8 µg aluminium hydroxide (Alhydrogel®, AlOH) per µg of R0.6C and stored at 2–8 °C. The study product was manufactured under current Good Manufacturing Practices [16] by Statens Serum Institut (Denmark) and vialed at Baccinex (Switzerland). Matrix-M1™ (hereafter referred to simply as Matrix-M) is a saponin-based adjuvant manufactured by Novavax AB, Sweden [19, 20].

### Study procedures

**Fig. 1:**
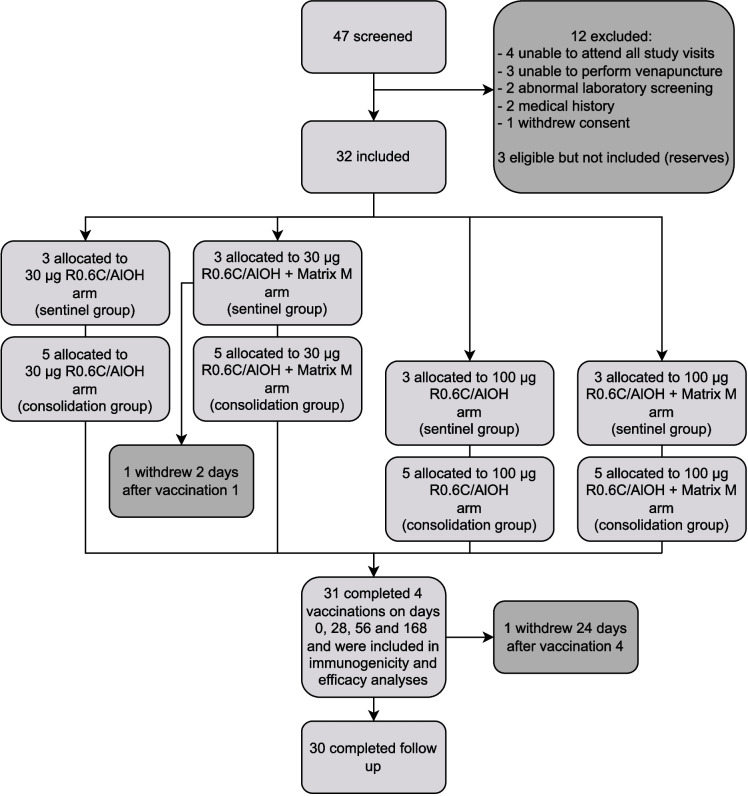
Screening and enrolment

Thirty-two participants were enrolled to receive four vaccinations intramuscularly in the deltoid muscle on alternating sides on days 0, 28, 56 and 168. Participants of childbearing potential were instructed to use adequate contraception throughout the study period. Participants were divided over the four study arms that received either 30 μg or 100 μg of R0.6C, each either adjuvanted with AlOH alone or combined with Matrix-M; *n* = 8 per R0.6C dose and adjuvant combination (Fig. 1). In order to maintain the same ratio of AlOH to Matrix-M adjuvants, 30-μg R0.6C/AlOH doses were admixed with 15-µg Matrix-M and 100-μg R0.6C/AlOH doses were admixed with 49-µg Matrix-M. The volume of the administered study products ranged from 0.15 mL to 0.63 mL depending on dose and adjuvant. A sentinel enrolment group of 3 participants in each study arm started vaccinations. A minimum of 7 days after the first vaccination in the sentinel groups of the two 30-μg R0.6C study arms, an interim assessment for any dose-related safety concerns was conducted by an independent safety monitoring committee, before the remaining 5 participants in each 30-μg R0.6C study arm started vaccinations (consolidation enrolment group) and before escalation to the 100-µg R0.6C dose. The same procedure with a sentinel enrolment group of 3 participants in each 100-μg R0.6C study arm, followed by a consolidation enrolment group of 5 participants in each study arm, was done for the 100 µg vaccinations. Adverse events (AEs) were collected at each study visit from the first vaccination until 84 days after the fourth vaccination. Blood samples for mosquito feeding assays and antibody measurements were taken at predefined timepoints throughout the study.

### Randomisation

Participants were randomly allocated 1:1 to receive AlOH alone or AlOH + Matrix-M using a Mersenne-Twister random number generator implemented in R. Randomisation was stratified on R0.6C dose and sentinel/consolidation group. Participants and study personnel were all aware of allocation (dose/adjuvant combination).

### Safety assessment

Per protocol, AEs were graded as mild/grade 1 (easily tolerated), moderate/grade 2 (interfering with daily activity), or severe/grade 3 (preventing daily activity), and in the case of fever as grade 1 (38.0–38.4 °C), grade 2 (38.5–38.9 °C) or grade 3 (≥ 39 °C). Additionally, local AEs are reported here according to the Food and Drug Administration (FDA) AE grading scale [21]. AEs were categorized by the International Classification of Diseases 10 code. Until 7 days after each vaccination, the following local adverse events were solicited: pain, pruritus, swelling, induration, and erythema at the injection site. Until 14 days after each vaccination, the following systemic adverse events were solicited: fever, headache, myalgia, fatigue, chills, and rash. Any other adverse events were categorized as unsolicited adverse events. For each adverse event, causality to the study procedures was categorized as not related, unlikely related, possibly related, probably related, or definitely related; where a dichotomous classification was required, the first two categories were together considered as unrelated and the latter three as related. Safety blood tests including haematology and biochemistry evaluations were performed at each study visit, except at the study visits 56 days after the third and fourth vaccinations.

### Quantification of vaccine-specific IgG concentrations

Serum concentrations of IgG antibodies against R0.6C and 6C antigens were quantified by enzyme-linked immunosorbent assays (ELISAs) [16, 22]. Nunc MaxiSorp™ 96-well plates (ThermoFisher) were coated overnight at 4 °C with 100 µl of 0.5 µg/mL antigen per well. Plates were blocked with 5% skimmed milk in PBS and subsequently incubated with diluted participant serum. Detection was done with 1:40,000 dilution goat anti-human IgG HRP (Invitrogen, Cat. No. 31412). Plates were developed by adding 100 µL tetramethylbenzidine and stopped with 50-µL 0.2M H2SO4. Absorbances were read at 450 nm on an iMark™ microplate absorbance reader (Bio-Rad). Analyses were performed using Auditable Data Analysis and Management System for ELISA (ADAMSEL FPL v1.1). Serially diluted TB31F monoclonal antibody with known concentration served as a standard curve. The standard curve was plotted on a logarithmic scale and fitted to a power trend line (R2 > 0.99) and optical density measurements for each test sample (average of duplicates that were no more than 25% different) were converted to concentrations in µg/mL relative to the standard curve. For each seroconverted participant, the ratio of 6C/R0.6C antibody concentrations at each study timepoint was also calculated.

### Purification of anti-R0.6C antibodies

Total IgG was purified using a 1 mL HiTrap(R) Protein G HP column (GE Healthcare) according to the manufacturer’s instructions with few modifications: 6 mL of total citrate plasma was diluted with 6-mL binding buffer (PBS) and precipitated with 12 mL 2X ammonium sulfate (100%) for 30 min at room temperature. The samples were centrifugated at 3200 × g for 15 min at room temperature and the pellet was resuspended in 24 mL of ammonium persulfate (50% saturated). A second centrifugation was performed at 16,100 × g for 10 min and the pellet was finally dissolved in 24 mL PBS. The samples were filtered using a 0.45-µm filter before loading on to the HiTrap® Protein G HP column. After loading the samples, the column was washed with 15 mL of binding buffer. Total IgG was eluted with 1 mL fractions IgG elution Buffer (Thermo scientific, [23]) in tubes containing 150-µL 1.0M Tris pH 8.8. Fractions containing IgG were pooled and buffer exchanged to 6 mL PBS using Vivaspin(R) 20 (30kDa MWCO) concentrators (Sartorius VS2022, Stonehouse, UK).

Anti-R0.6C antibodies were purified using n 1-mL HiTrap N-hydroxysuccinimide activated HP column (GE Healthcare) to which R0.6C was covalently coupled following the manufacturer’s instructions [16]. Total IgG, purified from participant serum, was loaded on the column followed by a wash step with 15 mL of PBS. Bound anti-R0.6C antibodies were eluted with 6 mL IgG elution Buffer (Thermo scientific, [23]) in tubes containing 150-µL 1.0M Tris pH 8.8. Fractions containing anti-R0.6C antibodies were pooled and buffer exchanged to 200 µL of 25% PBS using Vivaspin 20 (30kDa MWCO) concentrators. The final volume of samples containing R0.6C-specific antibodies thus equal 1/30th of the original plasma volumes.

### Mosquito feeding experiments

SMFAs were used to determine the TRA of participants’ sera and of affinity-purified anti-R0.6C antibodies, as described previously [24]. In short, 90 μL of participant’s serum or 90 μL R0.6C antibodies added to 90 μL freeze dried fetal calf serum (FCS), was mixed with 150 μL packed red blood cells and cultured *P. falciparum* NF54 gametocytes, and 30 μL naïve human serum containing active complement, before feeding to *Anopheles stephensi* (Sind-Kasur Nijmegen strain) mosquitoes. After 6–8 days, oocysts were counted in 20 fully-fed mosquitoes per feeding condition. TRA for participant sera was calculated as the reduction in oocysts compared to the participant’s pre-immunisation control serum. Affinity-purified R0.6C-specific IgGs were tested in two independent SMFA experiments using FCS as a negative control.

### Statistical analysis

Analyses were performed using SPSS V25 (IBM) and Graphpad Prism(R) V9.0.0. For comparison between study groups, the Mann–Whitney *U* test was used. Paired comparisons were performed with the Wilcoxon signed ranks test. Two-sided *p*-values < 0.05 were considered significant; Bonferroni correction for multiple testing was used where appropriate and as indicated. Individual level TRA of affinity purified R0.6C-specific IgGs was estimated using a mixed effects negative binomial regression model available as online data analysis tool [25].

## Results

### Recruitment and study population

Thirty-two malaria-naïve adults were enrolled sequentially to the study arms receiving 30 µg R0.6C per dose (*n* = 16 total) and 100 µg R0.6C per dose (*n* = 16 total). Stratified randomisation (i.e. *within* each sentinel and consolidation group) resulted in balanced baseline characteristics between participants immunised with R0.6C/AlOH only or with R0.6C/AlOH + Matrix-M (Table 1) for both the low and high doses. Overall, 66% of the participants was female, the mean age of participants was 28 years (range 18–53) and their mean BMI was 23.2 kg/m^2^ (range 18.7–29.3). One participant in the 30-µg R0.6C/AlOH + Matrix-M arm withdrew from the trial 2 days after their first vaccination, on the grounds of a hematoma resulting from venipuncture for routine safety blood collection; this AE was classified as mild and related to study procedures in general, but not to the investigational product itself. This participant is included in the safety analyses, but not in the immunological or functional analyses as no blood samples were collected for these endpoints. A second participant withdrew from the trial 24 days after receiving their fourth vaccination due to personal circumstances unrelated to the trial. All 30 other participants received all four vaccinations and completed follow-up (Fig. 1).

**Table 1:** Baseline characteristics

	**30 µg R0.6C/AlOH**	**30 µg R0.6C/AlOH + Matrix-M**	**100 µg R0.6C/AlOH**	**100 µg R0.6C/AlOH + Matrix-M**
**Participants (*n*)**	8	8	8	8
**Female/male (*n*)**	5/3	4/3	6/2	6/2
**Age (years)**	23 (18–51)	25 (19–53)	22 (20–47)	22 (18–49)
**Weight (kg)**	73.5 (57.0–90.0)	73.7 (61.0–75.6)	68.4 (57.8–89.0)	65.0 (57.0–72.8)
**BMI (kg/m**^**2**^**)**	24.2 (20.8–28.8)	22.2 (18.7–27.4)	23.1 (19.5–29.3)	22.6 (19.2–24.6)

Median values for age, weight and BMI at baseline are shown with the full range in brackets

### Safety and tolerability

**Fig. 2:**
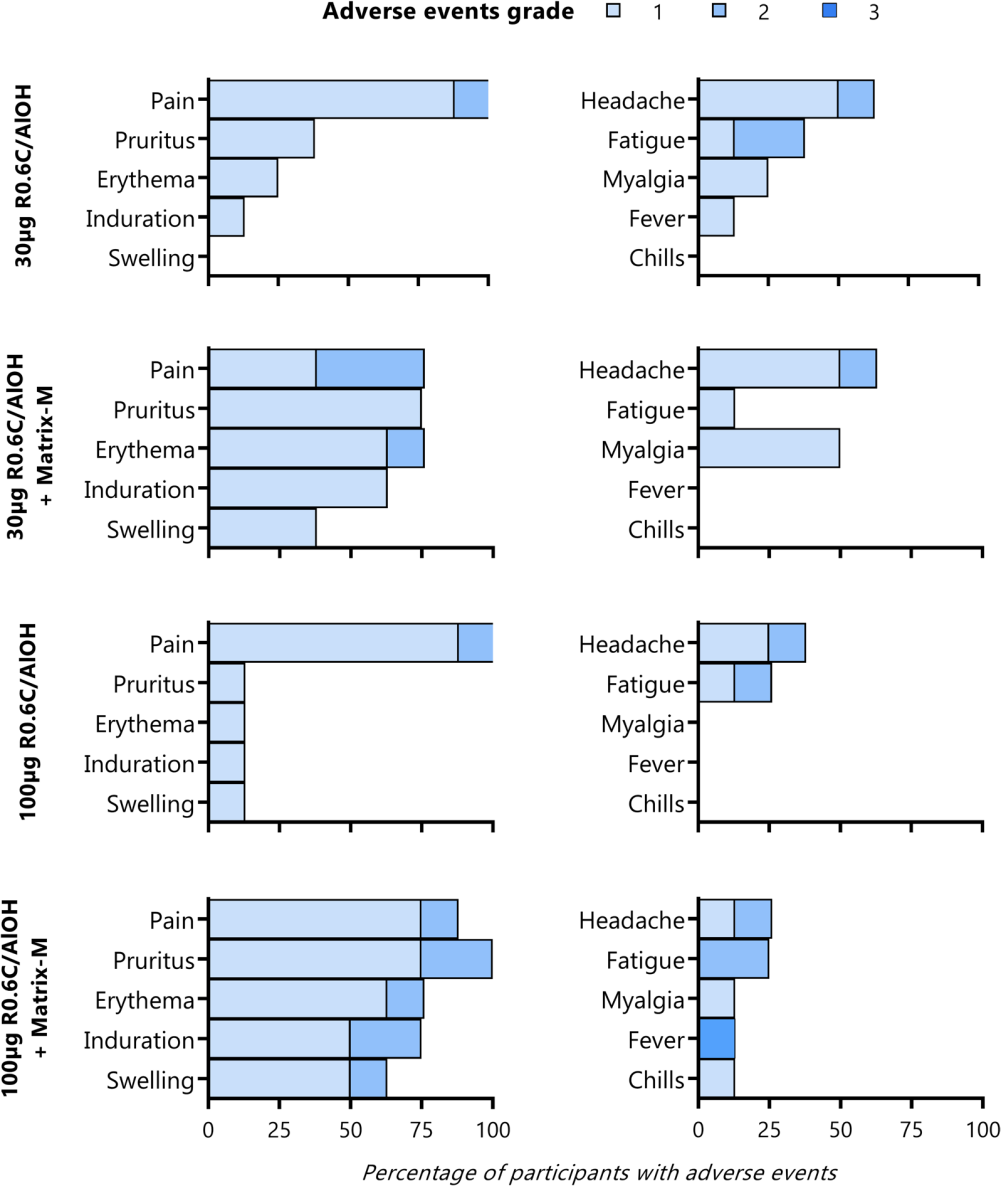
Solicited local and systemic adverse events. Per protocol, adverse events were graded as mild/grade 1 (easily tolerated), moderate/grade 2 (interfering with daily activity), or severe/grade 3 (preventing daily activity), and in the case of fever as grade 1 (38.0–38.4 °C), grade 2 (38.5–38.9 °C) or grade 3 (≥ 39 °C). Post hoc, local adverse events were also graded according to the Food and Drug Administration (FDA) Adverse Event grading scale (Supplementary Fig. 2, Additional file 1), to more conservatively reflect observed local reactogenicity. If there was more than one episode per participant, the highest grade adverse event was listed. *One participant withdrew from follow-up after the first immunisation and adverse events for this participant were recorded only until 2 days after the first immunisation. The solicited systemic adverse event ‘rash’ was not reported during the study

No serious adverse events occurred. Solicited adverse events were mostly local and mostly mild or moderate (Fig. 2; Additional file 1: Table S1, Fig. S1–S2). Fourteen out of 31 participants experienced some level of local reactogenicity after vaccinations #2, #3 and/or #4, consisting of erythema and induration or swelling (up to 22 cm in diameter), pruritus and/or pain. With the exception of one participant in the R0.6C/AlOH arm with mild symptoms, this local reactogenicity occurred only in participants who received vaccinations with R0.6C/AlOH + Matrix-M. Per protocol, these adverse events were graded mild or moderate based on disruption of daily activities, and resolved spontaneously within 3–4 days after onset. According to the more conservative current FDA AE severity grading scale [21], five of these individual occurrences would be classified as grade 3, based on the diameter of erythema, induration and/or swelling. Notably, although local reactogenicity was observed only following the second or later vaccinations, there was no indication of increased reactogenicity upon subsequent vaccinations in a given participant. Only one systemic grade 3 adverse event occurred: a participant-reported 39.4 °C fever the second night after the fourth vaccination in the 100 µg R0.6C/AlOH + Matrix-M arm, which was considered probably related to vaccination and had resolved spontaneously by the next morning. There were nine laboratory abnormalities considered clinically significant, mostly eosinophilia, that accompanied the local reactions and resolved spontaneously (Additional file 1: Table S2).

### Immunogenicity

**Fig. 3:**
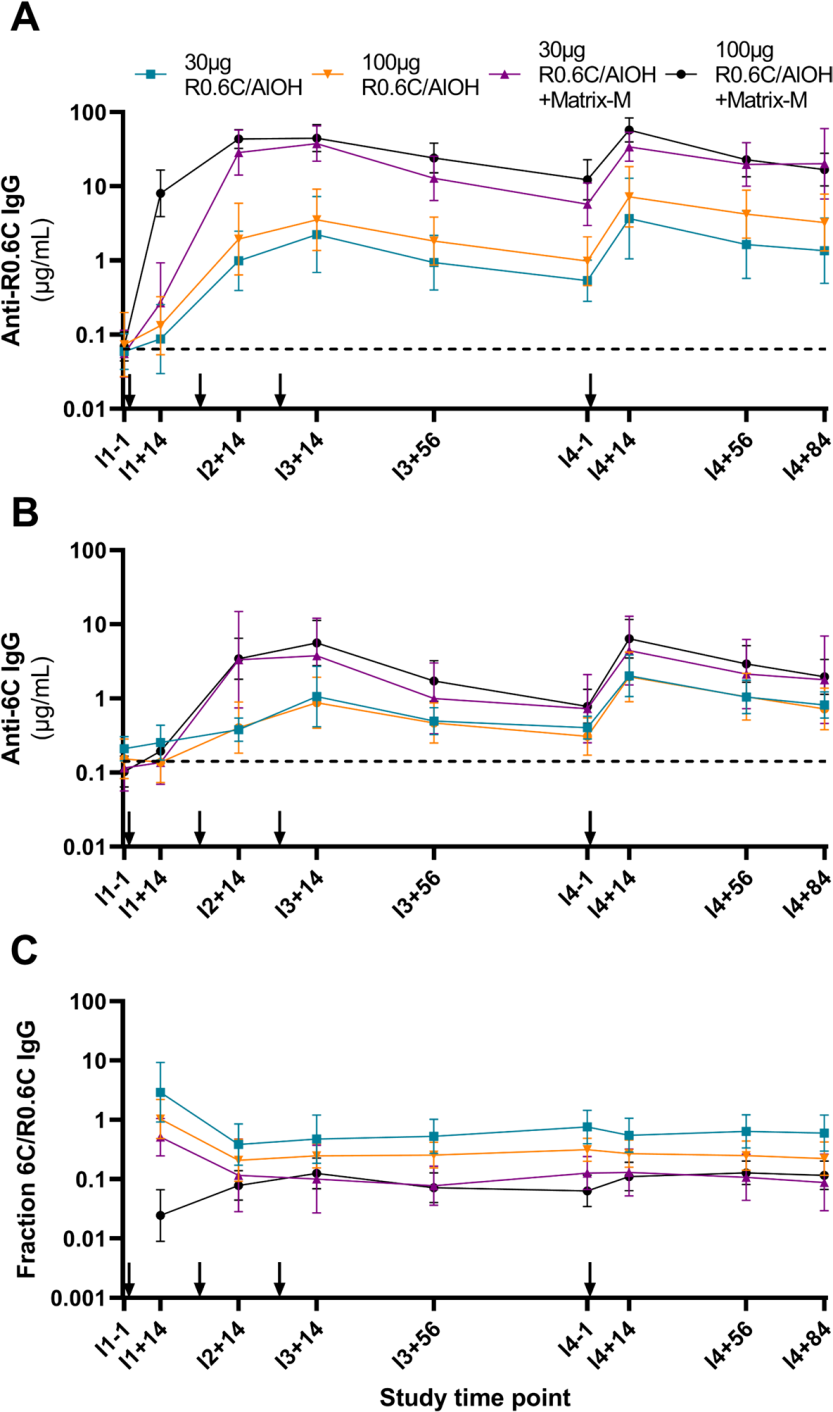
Anti-6C and anti-R0.6C IgG antibody responses over time. **A** Geometric mean anti-R0.6C antibody concentrations per study arm. **B** Geometric mean anti-6C antibody concentrations per study arm. **C** Geometric mean IgG responses against 6C as a fraction of total IgG responses against R0.6C per study arm. IgG concentrations in A and B are calculated using serially diluted anti-6C antibody TB31F of known concentration as a reference. Arrows represent vaccinations. Error bars indicate 95% CI. Dashed lines indicate the geometric mean of pre-immunisation (baseline) serum samples

Antibody responses against R0.6C and 6C recombinant proteins were detectable in all vaccinated participants, with the exception of one participant who received vaccinations with 30 µg R0.6C/AlOH (Fig. 3A, B; Additional file 1: Fig. S3A). Anti-6C IgG concentrations were significantly (approximately ten-fold) higher in the R0.6C/AlOH + Matrix-M study arms compared to the R0.6C/AlOH study arms on days I2 + 14 (*p* < 0.001), I3 + 14 (*p* < 0.001), I3 + 56 (*p* = 0.001), I4 + 14 (*p* = 0.002) and I4 + 56 (*p* = 0.006) after Bonferroni-correction for multiple comparisons (*p*-values of < 0.00625 were considered statistically significant), but not on days I1 + 14, I4 − 1 and I4 + 84. Anti-R0.6C IgG concentrations were significantly higher in the R0.6C/AlOH + Matrix-M study arms compared to the R0.6C/AlOH study arms on days I1 + 14, I3 + 14, I3 + 56, I4 − 1, I4 + 14, I4 + 56 and I4 + 84 (*p* < 0.001 for all timepoints) but not on I2 + 14. Within adjuvant groups, there was no significant difference in anti-6C or anti-R0.6C IgG concentrations between the 30 µg and 100 µg study arms at any timepoint after correction for multiple comparisons. Geometric mean IgG concentrations against both 6C and R0.6C induced by immunisation with R0.6C/AlOH alone increased somewhat after each subsequent immunisation. In contrast, IgG responses induced by R0.6C/AlOH + Matrix-M reached peak concentrations after only two immunisations and did not reach significantly higher concentrations after either the third or fourth immunisation (repeated measures ANOVA followed by pairwise comparison with Bonferroni correction). IgG responses against 6C as a fraction of total IgG responses against R0.6C remained stable over time in each dose/adjuvant arm from 14 days after the second immunisation onward. The geometric mean fraction was 10% in sera from participants immunised with R0.6C/AlOH + Matrix-M and significantly higher (geometric mean 40%; *p* < 0.001) in participants immunised with R0.6C/AlOH alone (Fig. 3C). Comparison of the slopes of regression lines from log transformed R0.6C and 6C IgG concentrations after the third and fourth vaccination revealed no significant differences in antibody decay rate (*p* = 0.86 and *p* = 0.89 respectively, Additional file 1: Fig. S4).

### Functional transmission-reducing activity

**Fig. 4:**
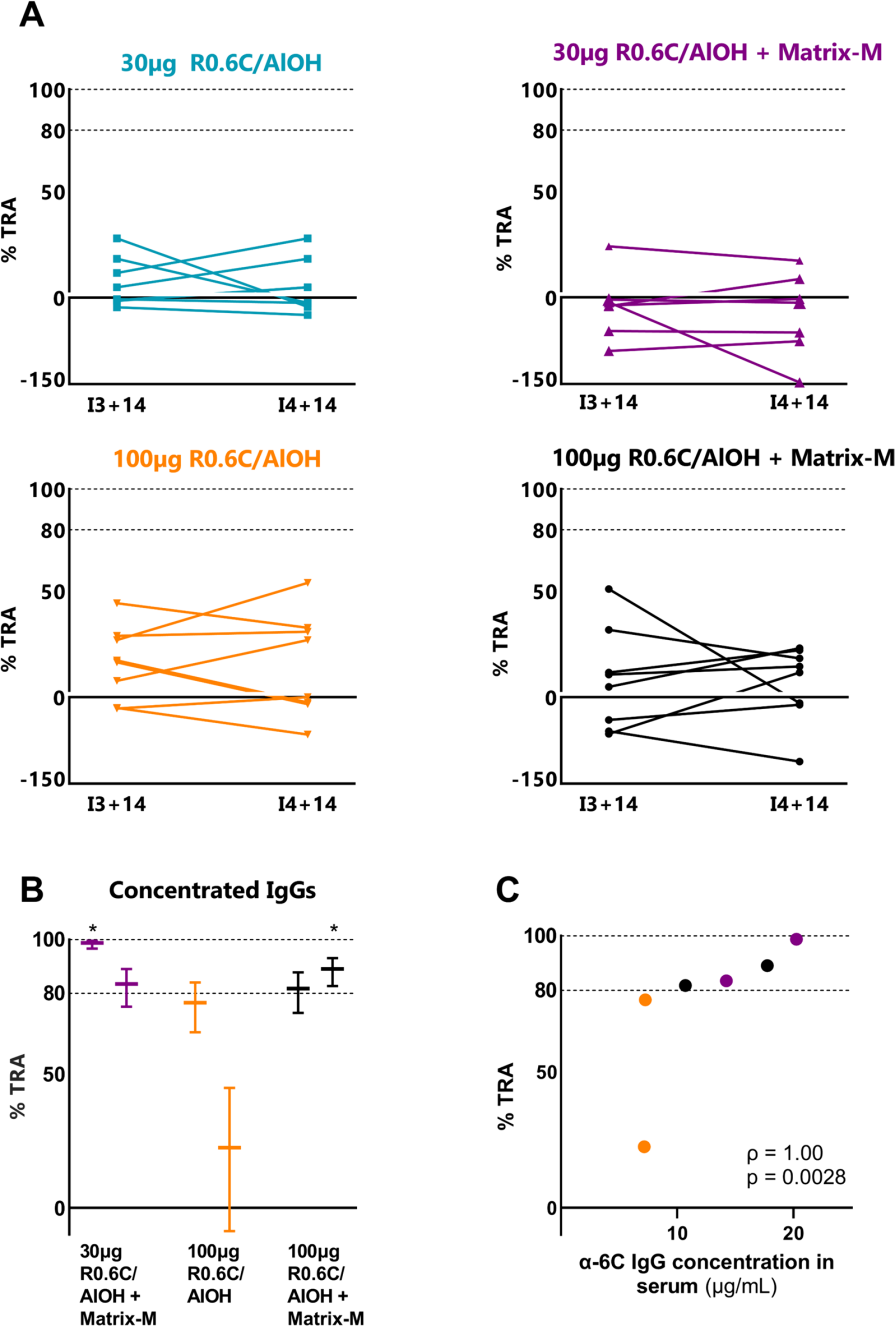
Functional transmission-reducing activity in SMFA. **A** SMFA with participants’ sera collected two weeks after the third and fourth immunisation. Each data point represents the transmission-reducing activity of participants’ sera compared to their pre-immunisation sera. **B** SMFA with purified and concentrated anti-R0.6C IgGs from sera of a selection of six participants collected at 2 weeks after the fourth immunisation. Two sera from the 30 µg R0.6C/AlOH + Matrix M, 100 µg R0.6C/AlOH and 100 µg R0.6C/AlOH + Matrix M arms were selected based on anti-6C antibody concentrations (ranging 7.2–20.3 µg/mL). **C** Correlation between anti-6C IgG serum concentrations and transmission-reducing activity of the concentrated IgGs purified therefrom (Spearman’s *ρ* = 1.00, *p* = 0.0028). Dashed lines at 80% indicate the predefined efficacy threshold of interest [4, 26]. Asterisks indicate TRA is statistically significantly higher than the threshold of 80%

No statistically significant reduction in oocyst density was seen in SMFA experiments using sera collected at 14 days after either the third or fourth immunisation from participants in any of the four study arms, as compared to their own baseline sera (Fig. 4A; Additional file 2). Since anti-6C IgG responses were detected in post-immunisation sera by ELISA, albeit at relatively low concentrations, additional SMFAs were performed post hoc with concentrated IgG from samples collected 14 days after the fourth immunisation from 6 participants with the highest such responses (*n* = 2 who received 100 µg R0.6C/AlOH with Matrix-M, *n* = 2 who received 100 µg R0.6C/AlOH alone and *n* = 2 who received 30 µg R0.6C/AlOH with Matrix-M). R0.6C-specific antibodies were purified on R0.6C-coated columns and concentrated to approximately 30 times the original volume. These samples were tested in two independent SMFA experiments and had estimated TRAs of 22 till 99% (Fig. 4B; Additional file 2). 6C IgG serum titres were associated with TRA (Spearman’s *ρ* = 1.0, *p* = 0.0028, Fig. 4C).

## Discussion

In this first-in-human study of a Pfs48/45-based *P. falciparum* transmission-blocking vaccine we show that vaccination with R0.6C/AlOH with or without Matrix-M is safe, immunogenic and induces functional Pfs48/45-specific transmission-blocking antibodies. While participants’ sera did not directly achieve TRA, concentrated anti-R0.6C IgGs purified from selected participants’ sera following vaccination exhibited strong TRA in SMFA. Together these data prove the concept of a 6C-based vaccine, but show that induced serum antibody concentrations were too low to directly confer TRA.

Although vaccination with R0.6C was generally well tolerated, the addition of Matrix-M to the R0.6C/Alhydrogel formulation induced more pronounced local reactogenicity, a finding that is likely associated with this adjuvant’s enhancing effect on R0.6C immunogenicity. As observed in pre-clinical rodent studies, the addition of a second adjuvant Matrix-M to the vaccine formulation significantly increased R0.6C and 6C antibody concentrations [16]. Moreover, in contrast to R0.6C/AlOH alone, antibody responses induced by R0.6C/AlOH + Matrix-M peaked after already the second vaccine dose rather than the fourth. However, although anti-6C IgG concentrations induced by R0.6C/AlOH + Matrix-M were still significantly higher than those induced by R0.6C/AlOH alone, anti-6C IgG as a fraction of total anti-R0.6C IgG was relatively lower in R0.6C/AlOH + Matrix-M sera, suggesting that the addition of Matrix-M adjuvant preferentially favours responses to the GLURP R0 fragment, which has been shown to be immunodominant in other fusion-protein vaccines [27].

It can be inferred that the addition of Matrix-M effectively accelerates the response to R0.6C, rapidly reaching a saturation point unattainable without Matrix-M; this response nevertheless remains insufficient to directly exert TRA, suggesting a likely impediment within the R0.6C construct itself. Furthermore, in contrast to several other malaria vaccines [28–31], the delayed fourth dose did not significantly increase vaccine-specific IgG responses in the R0.6C/AlOH with Matrix-M study arms. While we cannot rule out that subsequent vaccine doses with either adjuvant combination may have resulted in enhanced affinity maturation, it remains a valid consideration that such maturation could have been inadequate or targeted towards non-protective epitopes of 6C. Consequently, this might have led to an inability to elicit a directly measurable functional response in sera.

There are a number of interesting observations in our current vaccine trial that are of relevance for future vaccine optimization. Firstly, IgG responses against 6C made up only 10–40% of total IgG responses against R0.6C, indicating that the greatest fraction of induced antibodies target the non-functional R0 fragment. The fraction of 6C/R0.6C IgGs was roughly concordant with the relative size of the 6C fragment compared to the overall R0.6C fusion protein [17]. Secondly, the modest antibody responses were not dependent on the vaccine dose in the tested dose range, as antibody concentrations resulting from 30 µg and 100 µg R0.6C vaccine doses with a given adjuvant combination were similar. A clinical trial involving TB31F, a potent humanised monoclonal antibody that binds a conserved epitope on the 6C fragment of Pfs48/45, was recently conducted [12]. The study showed that the concentration of TB31F reaching 80% TRA, a threshold historically used to support clinical development of TBV, was determined to be 2.1 µg/mL (95% CI 1.9–2.3) [14]. This is lower than the anti-6C IgG concentrations that were induced in our current study (geometric mean 5.4 µg/mL in Matrix-M groups, SD 6.0). A likely explanation for this discrepancy could be that the total anti-6C IgG concentrations measured in our sera represent a polyvalent response of antibodies with different potencies, as we have previously identified human mAbs against 6C that differ in potency [10]. Compared with other subunit vaccines, where antigen-specific antibody concentrations of > 100 µg/mL are not exceptional [32, 33], the anti 6C-antibody levels induced in this study are modest.

Our functional results contrast with findings in pre-clinical animal models. Whereas we observed functional activity only using concentrated IgG but not directly in human sera, antibody responses in (unconcentrated) sera from animals were sufficient to induce > 99% TRA in SMFA [15, 34]. This discordance may be attributed to several factors, including interspecies differences in the immune system and immunological experience of a human adult compared with a laboratory mouse. Additionally, disparities in antigen presentation between animal models and humans may underlie differences in the quality and specificity of the elicited antibody responses [35, 36]. Nevertheless, our results suggest that up to 1–2 orders of magnitude higher anti-6C titres would be needed to achieve substantial transmission-reducing activity. Immunisations with an optimised vaccine formulation might achieve such sufficiently high anti-6C antibody concentrations. A more immunogenic vaccine delivery platform could be assessed, such as an mRNA vaccine [37, 38] or virus-like particles, as these were shown to enhance antibody responses in mice against the TBV candidate Pfs25 [39, 40]. Additionally, evaluation of R0.6C in an endemic setting may demonstrate superior antibody induction due to boosting of naturally acquired antibodies.

The greatest part of the total IgG response against R0.6C targets the non-functional R0 domain, which was included in the R0.6C construct to achieve the correct conformation of 6C during expression in *L. lactis*. Replacing the R0 domain with another 6C-stabilising component that is either less immunodominant, or that itself also induces transmission-blocking antibodies, could thus be beneficial. One promising candidate is the Pro domain of Pfs230, another antigen known to be the target of both naturally occurring and vaccine-induced transmission-blocking antibodies [41]. The functional domains of Pfs230 and Pfs48/45 have been fused with a linker sequence derived from CSP in the ProC6C construct, which was shown to elicit high titres of functional transmission-blocking antibodies in mice [34]. Two phase 1 trials [42] are currently evaluating the safety and immunogenicity of this TBV candidate, adsorbed to AlOH and formulated with and without Matrix-M adjuvant, in malaria-endemic populations in Burkina Faso (PACTR202201848463189) and Mali (ISRCTN13649456). An alternative strategy is to enhance the stability and biophysical properties of Pfs48/45-6C by combinatorial structure-based engineering of the Pfs48/45 antigen, to better focus the immune response against protective epitopes. This approach has been demonstrated to increase the transmission inhibitory capacity of sera by 1–2 orders of magnitude in rodents across three vaccine platforms, compared to the wild-type antigen [43], although this has not yet been assessed in humans.

Furthermore, it should be noted that it remains uncertain how TRA, as measured in SMFA, relates to true transmission reduction under field conditions. Due to its relatively high numbers of gametocytes in each blood meal that need to be neutralized, the SMFA might be too stringent, leading to an underestimation of the vaccine efficacy.

This study has several limitations. Firstly, the sample size was small and the study was not blinded. Although this is common in phase 1 trials, it is conceivable that this may have biased the reporting of adverse events. Secondly, the study was carried out during the SARS-CoV-2 pandemic; as a consequence, concurrent (suspected) SARS-CoV-2 infections led to postponement of follow-up visits outside the protocol-defined window on 7 occasions and to minor postponement of the third or fourth vaccination in four participants. The impact of postponing these follow-up visits was considered to have negligible impact on the safety and immunogenicity. In all instances, the greatest post-vaccination ‘risk-window’ had already passed, and the relatively short delay, when viewed in the context of the overall immunisation schedule timelines, is expected to exert, at most, a minor and immunologically insignificant impact thereon.

## Conclusions

We conclude that vaccination with R0.6C/AlOH with or without Matrix-M is safe and immunogenic, but the induced serum antibody titres were insufficient to achieve the threshold for *P. falciparum* transmission reduction in a malaria naïve population. Purified and concentrated anti-R0.6C IgGs were nevertheless able to induce up to 99% TRA, demonstrating for the first time the functionality and transmission-blocking potential of antibodies induced by a Pfs48/45-based vaccine.

### Supplementary Information

**Additional file 1: Table S1.** Solicited local and systemic adverse events. **Table S2.** Clinically significant laboratory abnormalities. **Figure S1.** Solicited local adverse events after each vaccination. **Figure S2.** FDA graded local adverse events. **Figure S3.** Individual level antibody responses. **Figure S4.** Antibody decay after vaccination 3 and 4.

**Additional file 2.** Mosquito-level SMFA data of neat serum and concentrated IgG’s.

## Data Availability

The datasets generated and analysed during the current study are available from the corresponding author on reasonable request.

## Abbreviations

AEs: Adverse events
AlOH: Aluminium hydroxide
CCMO: Central Committee on Research Involving Human Subjects
ELISA: Enzyme-linked immunosorbent assay
FCS: Fetal calf serum
FDA: Food and Drug Administration
IgG: Immunoglobulin G
PBS: Phosphate-buffered saline
SMFA: Standard membrane feeding assay
TBV: Transmission-blocking vaccine
TRA: Transmission-reducing activity
