# Supplementary material for: A Pfs48/45-based vaccine to block Plasmodium falciparum transmission: phase 1, open-label, clinical trial
Source: BMC Med. 2024 Apr 23;22:170. doi: 10.1186/s12916-024-03379-y (PMC11036667; doi:10.1186/s12916-024-03379-y)
Supplement: Supplementary file 1 — Additional file 1: Table S1. Solicited local and systemic adverse events. Table S2. Clinically significant laboratory abnormalities. Figure S1. Solicited local adverse events after each vaccination. Figure S2. FDA graded local adverse events. Figure S3. Individual level antibody responses. Figure S4. Antibody decay after vaccination 3 and 4. [file 12916_2024_3379_MOESM1_ESM.docx]

Additional file 1

Table of contents

[Table S1 2](#_Toc162424944)

[Table S2 4](#_Toc162424945)

[Figure S1 5](#_Toc162424946)

[Figure S2 6](#_Toc162424947)

[Figure S3 7](#_Toc162424948)

[Figure S4 8](#_Toc162424949)

Table S1 **– Solicited local and systemic adverse events**

|  |  | 30µg R0.6C/AlOH (n=8) | | 30µg R0.6C/AlOH + Matrix-M (n=8)* | | 100µg R0.6C/AlOH (n=8) | | 100µg R0.6C/AlOH + Matrix-M (n=8) | | All (n=32) | |
| --- | --- | --- | --- | --- | --- | --- | --- | --- | --- | --- | --- |
|  |  | #Subjects | % | #Subjects | % | #Subjects | % | #Subjects | % | #Subjects | % |
| Pain (M79.62) | Grade 1 | 7 | 88 | 3 | 38 | 7 | 88 | 7 | 88 | 24 | 75 |
|  | Grade 2 | 1 | 13 | 3 | 38 | 1 | 13 | 1 | 13 | 6 | 19 |
|  | Grade 3 | 0 | 0 | 0 | 0 | 0 | 0 | 0 | 0 | 0 | 0 |
| Pruritus (L29) | Grade 1 | 3 | 38 | 6 | 75 | 1 | 13 | 6 | 75 | 16 | 50 |
|  | Grade 2 | 0 | 0 | 0 | 0 | 0 | 0 | 2 | 25 | 2 | 6 |
|  | Grade 3 | 0 | 0 | 0 | 0 | 0 | 0 | 0 | 0 | 0 | 0 |
| Swelling (R22.9) | Grade 1 | 0 | 0 | 3 | 38 | 1 | 13 | 4 | 50 | 8 | 25 |
|  | Grade 2 | 0 | 0 | 0 | 0 | 0 | 0 | 1 | 13 | 1 | 3 |
|  | Grade 3 | 0 | 0 | 0 | 0 | 0 | 0 | 0 | 0 | 0 | 0 |
| Erythema (L53.8) | Grade 1 | 2 | 25 | 5 | 63 | 1 | 13 | 5 | 63 | 13 | 41 |
|  | Grade 2 | 0 | 0 | 1 | 13 | 0 | 0 | 1 | 13 | 2 | 6 |
|  | Grade 3 | 0 | 0 | 0 | 0 | 0 | 0 | 0 | 0 | 0 | 0 |
| Induration (R23.4) | Grade 1 | 1 | 13 | 5 | 63 | 1 | 13 | 4 | 50 | 11 | 34 |
|  | Grade 2 | 0 | 0 | 0 | 0 | 0 | 0 | 2 | 25 | 2 | 6 |
|  | Grade 3 | 0 | 0 | 0 | 0 | 0 | 0 | 0 | 0 | 0 | 0 |
| Any local AE* | Grade 1 | 8 | 100 | 7 | 88 | 8 | 100 | 8 | 100 | 31 | 97 |
|  | Grade 2 | 1 | 13 | 3 | 38 | 1 | 13 | 4 | 50 | 9 | 28 |
|  | Grade 3 | 0 | 0 | 0 | 0 | 0 | 0 | 0 | 0 | 0 | 0 |
|  |  |  |  |  |  |  |  |  |  |  |  |
|  | Any grade | 8 | 100 | 7 | 88 | 8 | 100 | 8 | 100 | 31 | 97 |
|  |  |  |  |  |  |  |  |  |  |  |  |
|  |  |  |  |  |  |  |  |  |  |  |  |
| Chills (R68.83) | Grade 1 | 0 | 0 | 0 | 0 | 0 | 0 | 1 | 13 | 1 | 3 |
|  | Grade 2 | 0 | 0 | 0 | 0 | 0 | 0 | 0 | 0 | 0 | 0 |
|  | Grade 3 | 0 | 0 | 0 | 0 | 0 | 0 | 0 | 0 | 0 | 0 |
| Fatigue (R53) | Grade 1 | 1 | 13 | 1 | 13 | 1 | 13 | 0 | 0 | 3 | 9 |
|  | Grade 2 | 2 | 25 | 0 | 0 | 1 | 13 | 2 | 25 | 5 | 16 |
|  | Grade 3 | 0 | 0 | 0 | 0 | 0 | 0 | 0 | 0 | 0 | 0 |
| Fever (R50.9) | Grade 1 | 1 | 13 | 0 | 0 | 0 | 0 | 0 | 0 | 1 | 3 |
|  | Grade 2 | 0 | 0 | 0 | 0 | 0 | 0 | 0 | 0 | 0 | 0 |
|  | Grade 3 | 0 | 0 | 0 | 0 | 0 | 0 | 1 | 13 | 1 | 3 |
| Headache (R51) | Grade 1 | 4 | 50 | 4 | 50 | 2 | 25 | 1 | 13 | 11 | 34 |
|  | Grade 2 | 1 | 13 | 1 | 13 | 1 | 13 | 1 | 13 | 4 | 13 |
|  | Grade 3 | 0 | 0 | 0 | 0 | 0 | 0 | 0 | 0 | 0 | 0 |
| Myalgia (M79.1) | Grade 1 | 2 | 25 | 4 | 50 | 0 | 0 | 1 | 13 | 7 | 22 |
|  | Grade 2 | 0 | 0 | 0 | 0 | 0 | 0 | 0 | 0 | 0 | 0 |
|  | Grade 3 | 0 | 0 | 0 | 0 | 0 | 0 | 0 | 0 | 0 | 0 |
| Rash (T88.1) | Grade 1 | 0 | 0 | 0 | 0 | 0 | 0 | 0 | 0 | 0 | 0 |
|  | Grade 2 | 0 | 0 | 0 | 0 | 0 | 0 | 0 | 0 | 0 | 0 |
|  | Grade 3 | 0 | 0 | 0 | 0 | 0 | 0 | 0 | 0 | 0 | 0 |
| Any systemic AE* | Grade 1 | 5 | 63 | 6 | 75 | 5 | 63 | 3 | 38 | 19 | 59 |
|  | Grade 2 | 2 | 25 | 1 | 13 | 2 | 25 | 2 | 25 | 7 | 22 |
|  | Grade 3 | 0 | 0 | 0 | 0 | 0 | 0 | 1 | 13 | 1 | 3 |
|  |  |  |  |  |  |  |  |  |  |  |  |
|  | Any grade | 6 | 75 | 6 | 75 | 5 | 63 | 4 | 50 | 21 | 66 |

Systemic and local solicited adverse events throughout the study. If there was more than one episode per participant, the highest grade adverse event was listed. *One participant withdrew from follow-up after the first immunisation and adverse events for this participant were recorded only until 2 days after the first immunisation.

Table S2 **– Clinically significant laboratory abnormalities**

|  |  | 30µg R0.6C/AlOH (n=8) | | 30µg R0.6C/AlOH + Matrix-M™ (n=8)* | | 100µg R0.6C/AlOH (n=8) | | 100µg R0.6C/AlOH + Matrix-M™ (n=8) | | All (n=32) | |
| --- | --- | --- | --- | --- | --- | --- | --- | --- | --- | --- | --- |
|  |  | #Subjects | % | #Subjects | % | #Subjects | % | #Subjects | % | #Subjects | % |
| Eosinophils (cell/mm3) | Grade 1 (650 - 1,500) | 0 | 0 | 2 | 25 | 0 | 0 | 1 | 13 | 3 | 9 |
|  | Grade 2 (1,501 - 5,000) | 0 | 0 | 0 | 0 | 0 | 0 | 0 | 0 | 0 | 0 |
|  | Grade 3 (>5,000) | 0 | 0 | 0 | 0 | 0 | 0 | 0 | 0 | 0 | 0 |
| Leukocytes (cell/mm3) | Grade 1 (10,800 - 15,000) | 0 | 0 | 0 | 0 | 0 | 0 | 0 | 0 | 0 | 0 |
|  | Grade 2 (15,001 - 20,000) | 0 | 0 | 0 | 0 | 0 | 0 | 1 | 13 | 1 | 3 |
|  | Grade 3 (20,001 - 25,000) | 0 | 0 | 0 | 0 | 0 | 0 | 0 | 0 | 0 | 0 |
| Hemoglobin (g/dL) | Grade 1 (11.0 - 12.0) | 0 | 0 | 0 | 0 | 0 | 0 | 0 | 0 | 0 | 0 |
|  | Grade 2 (9.5 - 10.9) | 0 | 0 | 0 | 0 | 1 | 13 | 0 | 0 | 1 | 3 |
|  | Grade 3 (8.0 - 9.4) | 0 | 0 | 0 | 0 | 0 | 0 | 0 | 0 | 0 | 0 |

Laboratory abnormalities throughout the study. Only abnormalities that were considered potentially clinically significant are depicted. If there was more than one episode per participant, the highest grade abnormality is listed. *One participant withdrew from follow-up after the first immunisation and blood samples for this participant were collected only until 2 days after the first immunisation.

Figure S1 **– Solicited local adverse events after each vaccination**


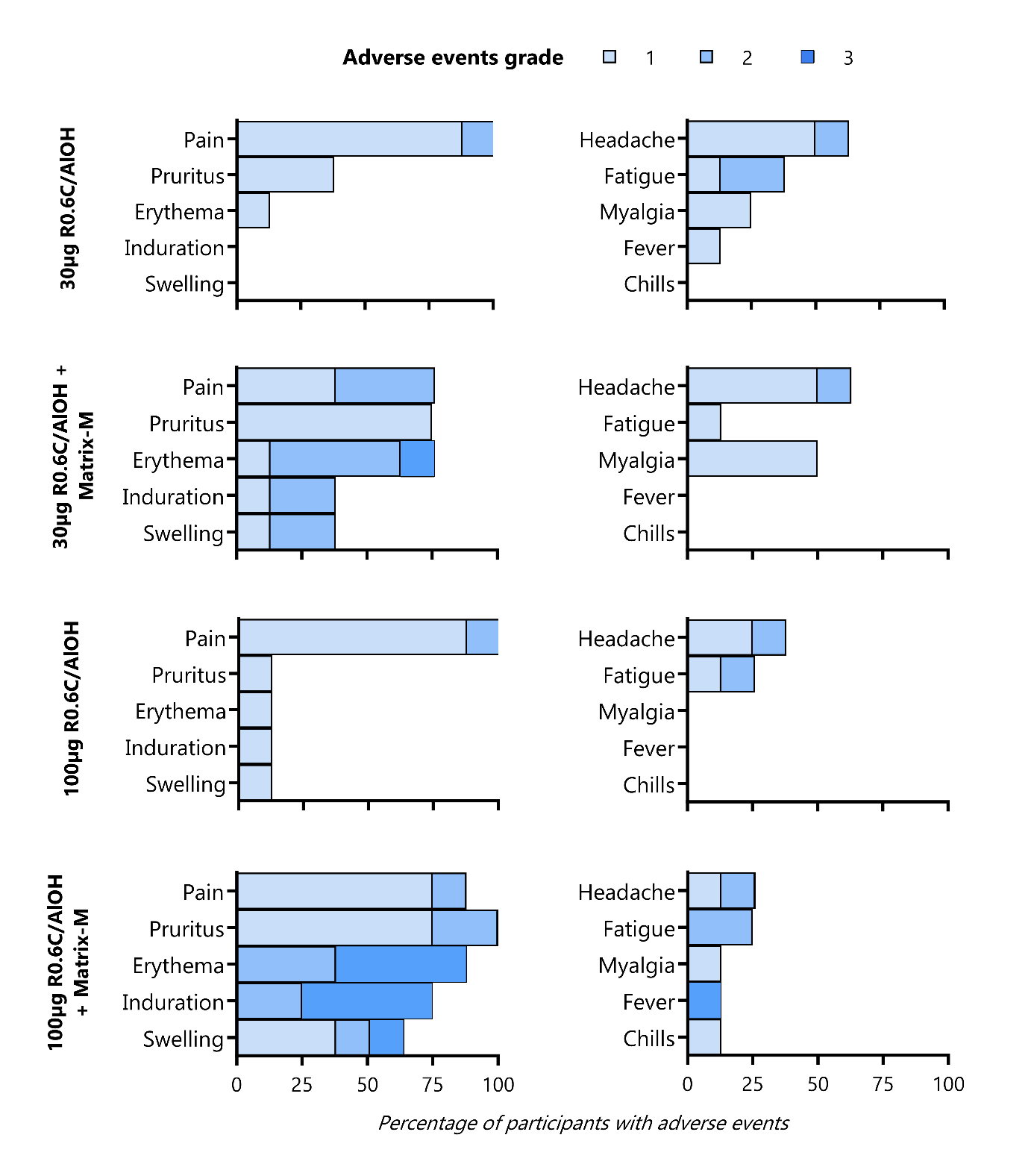


Figure S2 **– Solicited local and systemic adverse events graded according to the Food and Drug Administration (FDA) Adverse Event grading scale.**


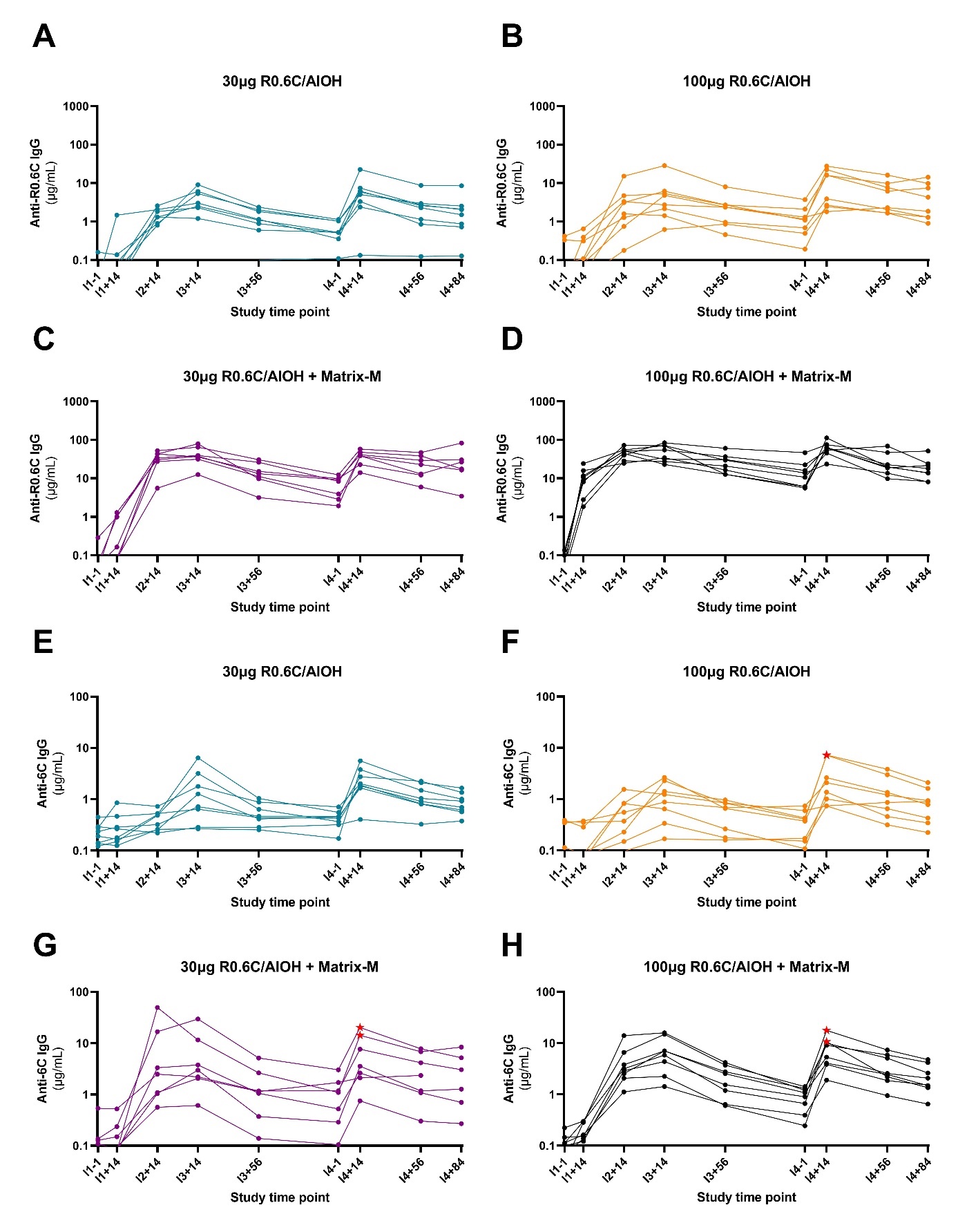


Figure S3 **– Individual level antibody responses.** Red stars in F), G) and H), indicate selected samples for IgG purification and concentration.


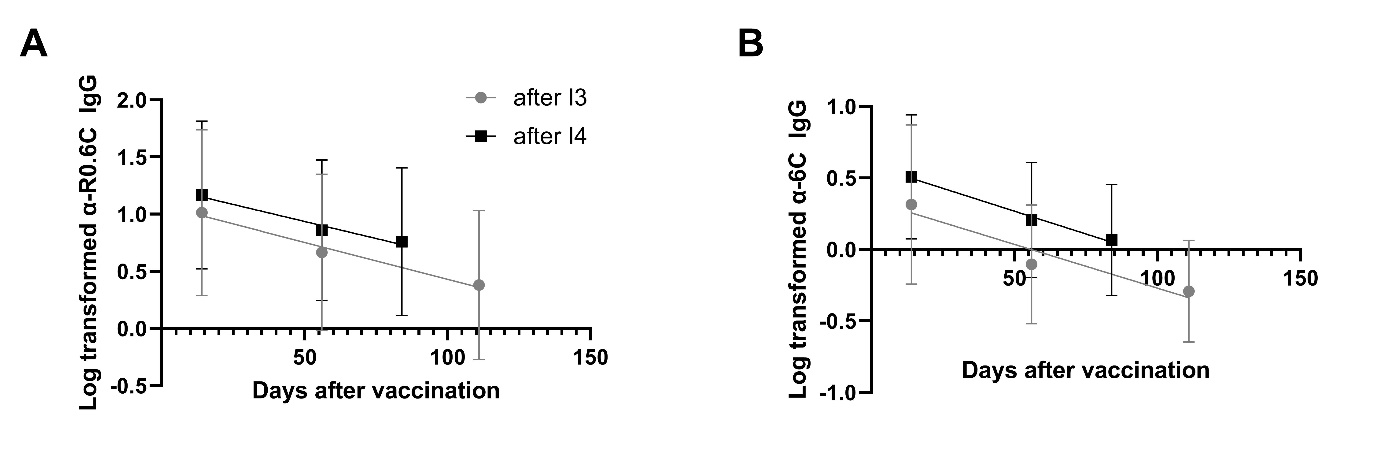


Figure S4 **– Antibody decay after vaccination 3 and 4.** Linear regression analysis on log transformed anti-R0.6C IgG (A) or anti-6C IgG concentrations (B) was performed to evaluate the antibody decay rate after three vaccinations (after I3, gray dots) compared to after four vaccinations (after I4, black squares).
